# The inhibitory effect of gadolinium-conjugated diflunisal on platelet function through regulating outside-in signaling of αIIbβ3 integrin

**DOI:** 10.3389/fphar.2026.1936307

**Published:** 2026-10-19

**Authors:** Malk Eun Pak, Yeon-Ji Kim, Hado Choi, Garam Choi, Yeoun-Hee Kim, Kyungho Kim

**Affiliations:** 1 Korean Medicine-Application Center, Korea Institute of Oriental Medicine, Daegu, Republic of Korea; 2 Korean Convergence Medical Science Major, KIOM School, University of Science and Technology (UST), Daejeon, Republic of Korea; 3 Department of R & D Center, Etnova Therapeutics Corp., Suwon-si, Gyeonggi-do, Republic of Korea

**Keywords:** antithrombotic effect, cerebral ischemia, diflunisal, gadolinium, platelet activation, thrombosis

## Abstract

The gadolinium-conjugated diflunisal derivative ETN102 has previously been shown to suppress cyclooxygenase-2 (COX-2) levels, reduce glial activation, and attenuate neuronal damage associated with stroke. In the present study, we investigated whether ETN102 modulates platelet aggregation and activation. ETN102 selectively inhibited thrombin-induced platelet aggregation while sparing platelet responses to other agonists, including collagen, adenosine diphosphate (ADP), and U46619. ETN102 also reduced adenosine triphosphate (ATP) secretion under aggregating conditions without altering thromboxane B_2_ (TXB_2_) generation, suggesting that its antiplatelet effect is independent of the COX-1-dependent thromboxane signaling. Flow cytometric and biochemical analyses further demonstrated that ETN102 suppressed thrombin-induced αIIbβ3 integrin activation, platelet spreading, clot retraction, and the phosphorylation of focal adhesion kinase (FAK) and proline-rich tyrosine kinase 2 (Pyk2), without affecting granule secretion, intracellular Ca^2+^ mobilization, or phosphorylation of Syk, PI3K, and AKT signaling molecules. *In vivo*, ETN102 significantly prolonged carotid artery occlusion time in a ferric chloride (FeCl_3_)-induced thrombosis model and increased tail bleeding time and hemoglobin content. In addition, ETN102 reduced infarct volume and improved neuronal survival markers in a murine model of cerebral ischemia/reperfusion injury. These findings demonstrate that ETN102 is a thrombin-context selective modulator of platelet integrin αIIbβ3 outside-in signaling, with antithrombotic and neuroprotective potential.

## 1 Introduction

Platelets play a central role in hemostasis by rapidly initiating thrombus formation to prevent excessive bleeding after vascular injury. Beyond this physiological function, platelets also contribute to pathological thrombosis and are critically involved in cardiovascular disorders such as myocardial infarction, ischemic stroke, and thromboembolism (Koupenova et al., 2018). Advances in platelet biology have elucidated complex regulatory mechanisms governing platelet adhesion, activation, and crosstalk with endothelial and immune cells, thereby supporting the development of targeted antithrombotic therapies (Ni and Freedman, 2003). In addition to their hemostatic and thrombotic functions, platelets participate in inflammatory and immune processes and have been investigated as mediators and therapeutic platforms in tissue repair, immune regulation, and disease (Li et al., 2018; Zou et al., 2025). However, the molecular mechanisms that connect platelet-mediated inflammatory regulation to thrombotic outcomes remain incompletely understood, particularly with respect to signaling events downstream of classical agonist-induced activation pathways.

Nonsteroidal anti-inflammatory drugs (NSAIDs) are widely used to alleviate pain and inflammation through inhibition of prostaglandin synthesis (Moses and Bertone, 2002). Beyond their primary therapeutic application, NSAIDs confer additional medical benefits, including antithrombotic effects (Meek et al., 2010). These actions are mainly mediated through inhibition of cyclooxygenase (COX) enzymes, which convert arachidonic acid into prostaglandins. COX-1 is generally associated with constitutive physiological homeostasis, whereas COX-2 is more closely associated with inducible inflammatory responses (Faki and Er, 2021). In platelets, COX-dependent metabolism contributes to thromboxane A_2_ (TXA_2_) production, which promotes platelet activation, aggregation, and vasoconstriction. Importantly, platelet-derived TXA_2_ synthesis depends predominantly on COX-1, whereas COX-2 is more closely associated with inflammatory responses in anucleate platelets (Hu et al., 2017).

A previous study described ETN102, a gadolinium-conjugated diflunisal derivative developed as a magnetic resonance imaging contrast-related anti-inflammatory agent. ETN102 exhibited enhanced binding properties relative to diflunisal and suppressed COX-2 upregulation, glial activation, and neuronal damage through the attenuation of neuroinflammation (Kim et al., 2020). Despite these findings, the effects of ETN102 on platelet biology and thrombosis remain undefined. In particular, it is unclear whether ETN102 influences platelet function through conventional COX-dependent mechanisms or through alternative signaling pathways involved in platelet aggregation and thrombus stabilization.

In the present study, we investigated the effects of ETN102 on platelet function, activation, integrin signaling, thrombosis, and ischemic injury. Our data show that ETN102 selectively inhibits thrombin-induced platelet aggregation, αIIbβ3 integrin activation, platelet spreading, and clot retraction, while sparing platelet responses triggered by other agonists. Mechanistically, ETN102 did not broadly suppress proximal platelet activation signaling but preferentially impaired thrombin-associated αIIbβ3 integrin-mediated outside-in signaling, as reflected by reduced FAK and Pyk2 phosphorylation. Furthermore, ETN102 exerted antithrombotic effects *in vivo* and attenuated ischemic brain injury. These findings identify ETN102 as a context-selective modulator of platelet integrin signaling rather than a broad-spectrum inhibitor of platelet activation.

## 2 Materials and methods

### 2.1 Reagents

Human thrombin, PGE1, dimethyl sulfoxide (DMSO), adenosine diphosphate (ADP), fibrinogen, human fibrinogen, ferric chloride (FeCl_3_), Acetylsalicylic acid (ASA), 2, 3, 5-triphenyl tetrazolium chloride (TTC), and all reagents were purchased from Sigma (St. Louis, MO). Equine tendon collagen (type I) and ATP luciferin/luciferase reagent were obtained from Chrono-log (Havertown, PA). Phycoerythrin (PE)-conjugated isotype control IgGs, rat monoclonal antibodies against mouse P-selectin, and activated αIIbβ3 (JON/A) were from Emfret Analytics (Eibelstadt, Germany). Antibodies against phospho-Pyk2 at Tyr 402, phospho-FAK at Tyr397, phospho-Syk at Tyr525/526, phospho-PI3K p85α/β at Tyr458/Tyr199, phospho-Akt at Ser473, COX1, COX2, Total Pyk2, Total FAK, Total Syk, Total PI3K p85, Total AKT, and actin were obtained from Cell Signaling (Danvers, MA, United States). Calcium dye (FLIPR Calcium Assay kit) was from Molecular Devices (Sunnyvale, CA, United States).

### 2.2 Chemicals

ETN102 was obtained from Etnova Therapeutics Corp., and prepared to a purity of 99.7%, as referenced in the literature (Kim et al., 2020), with further modifications (Figure 1A). The molecular weight of ETN102 is 832.92 g/mol. Chromatographic analysis of ETN102 was performed using a high-performance liquid chromatography (HPLC) system equipped with a UV detector. A Phenomenex SynergiTM fusion RP 80 Å column (4.0 µm, 2.0 × 150 mm) was maintained at a column temperature of 40 °C, with a flow rate of 0.2 mL/min. The mobile phase consisted of water and methanol with 0.1% formic acid in 10 mM ammonium formate. The injection volume was 30 μL, and the UV detection was conducted at 254 nm. The purity data were acquired based on the following gradient system: Tmin/B%: T0/10, T5/10, T10/40, T40/60, T45/60, T50/10, T60/10 (Figure 1B).

**FIGURE 1:**
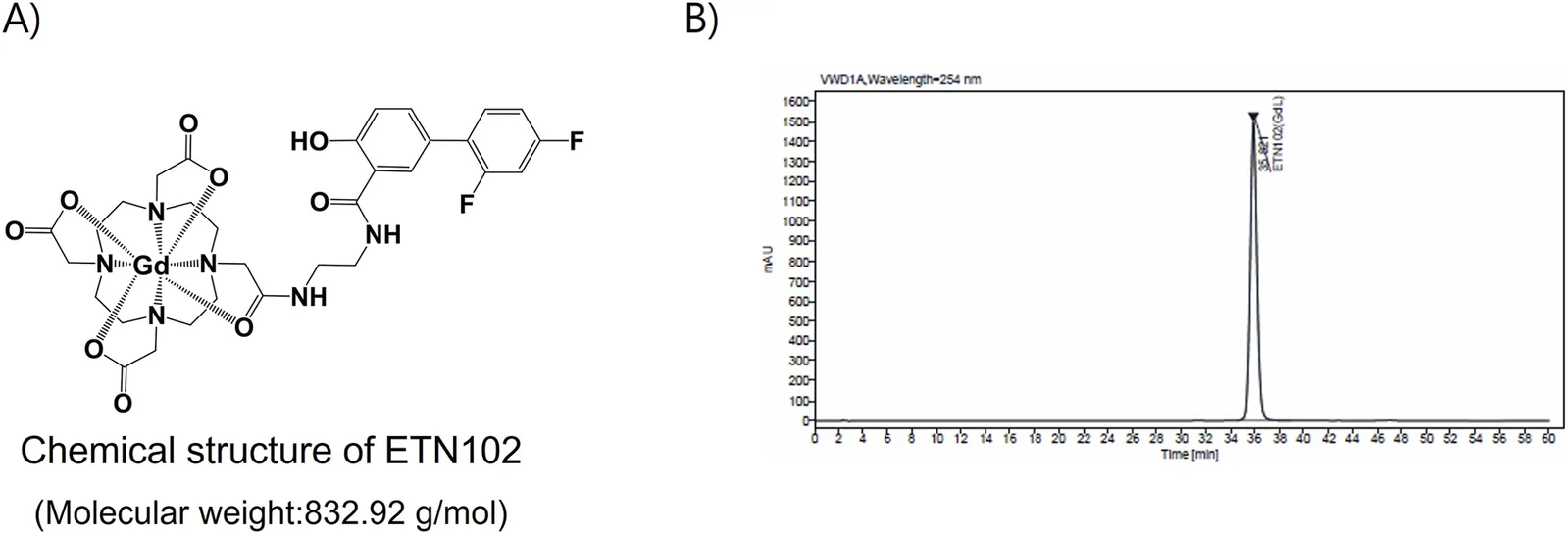
Chemical structure of ETN102, a gadolinium-conjugated diflunisal derivative. **(A)** The chemical structure of ETN102 (molecular weight, 832.92 g/mol) is depicted. **(B)** HPLC analysis data for ETN102 is presented, including a representative HPLC chromatogram recorded at 254 nm, where a prominent peak was observed at a retention time of 35.821 min. The main peak accounted for 99.71% of the total area, thereby confirming the high purity of ETN102.

### 2.3 Animals

Wild-type male C57BL/6J mice (6–8 weeks old, 18–22 g body weight (BW)) were purchased from DooYeol Biotech (Seoul, Korea) and acclimated for 1 week. Subsequently, the WT mice were randomly assigned to four groups, each comprising ten animals: (1) vehicle control group (administered vehicle), (2) low-dose of ETN102 treatment group (40 mg/kg BW), (3) high-dose of ETN102 treatment group (83 mg/kg BW), and (4) acetylsalicylic acid (ASA) treatment group (100 mg/kg BW). The animals were housed in a conventional animal facility with *ad libitum* access to food and water, maintained under controlled temperature and humidity conditions, and subjected to a 12-h light-dark cycle. All procedures were performed in accordance with the National Animal Welfare Law of Korea, and experimental protocols were approved by the Korea Institute of Oriental Medicine (KIOM, Daegu, Korea) Care Committee (reference number #D-22-092, #D-23-078). Animal anesthesia and euthanasia were conducted following the American Veterinary Medical Association (AVMA) Guidelines for the Euthanasia of Animals. Mice were anesthetized with isoflurane (Hana pharm co., Seoul, South Korea) using a calibrated vaporizer. For anesthesia induction, mice were placed in an induction chamber and exposed to 3% isoflurane delivered in 0.8 L/min of 100% oxygen. Once anesthetized, immobilization was maintained via a nose cone with 2% isoflurane in 0.8 L/min of oxygen. After blood collection, complete euthanasia was achieved by cervical dislocation to ensure death, in full compliance.

### 2.4 Platelet isolation

Mice were fully anesthetized using 3% isoflurane until the toe-pinch reflex, blood was obtained via venipuncture of the abdominal vein using a syringe pre-treated with an acid-citrate-dextrose solution (ACD, Sigma) for platelet isolation. Platelet-rich plasma (PRP) was obtained by centrifugation at 300 × g for 20 min at room temperature. Following PRP separation, prostaglandin E1 (PGE1) was added at a concentration of 0.5 µM to prevent platelet activation. The mixture was subsequently centrifuged at 700 × g for 4 min to remove residual blood cells, resulting in a platelet pellet. This pellet was washed with HEPES-Tyrode buffer containing 10% ACD and centrifuged again at 700 × g for 5 min. Finally, the platelet pellet was resuspended in HEPES-Tyrode buffer to a final concentration of 3 × 10^8^ cells/mL and utilized for experimental procedures.

### 2.5 Platelet aggregation

Mouse platelets were suspended in HEPES-Tyrode buffer (3 × 10^8^ cells/mL) and pre-incubated with ETN102 (100, 300, or 500 µM) or vehicle for 15 min at 37 °C. Platelet aggregation was induced using thrombin, collagen, ADP, and U46619 (an endoperoxide/thromboxane analog) and measured with a Chrono-Log aggregometer (Havertown, PA, United States) at 37 °C under stirring conditions (1,000 rpm).

### 2.6 ATP secretion

Mouse platelets were pre-incubated with varying concentrations of ETN102 (100, 300, and 500 µM) or vehicle control (distilled water) for 10 min at 37 °C. Subsequently, platelet activation was induced using thrombin, a known agonist in platelet aggregation, at 37 °C. Platelet adenosine triphosphate (ATP) secretion was assessed by quantifying ADP/ATP release through the addition of a luciferin/luciferase reagent (Chrono-log) to the platelet suspension. Measurements were conducted using a 4-channel platelet lumi-aggregometer (Chrono-Log Corp., Havertown, PA, United States) at 37 °C with stirring at 1,000 rpm. In some experiments, 2 mM EGTA was used to disrupt fibrinogen-αIIbβ3 interactions to distinguish primary agonist-induced secretion from integrin-dependent secondary amplification.

### 2.7 TXB_2_ generation assay

Washed platelets were pre-incubated with varying concentrations of ETN102 (100, 300, and 500 µM) or vehicle control (distilled water) for 10 min at 37 °C. Subsequently, platelet activation was induced by thrombin in a platelet aggregation system maintained at 37 °C. After 5 min, platelet activation was terminated by the addition of 0.1 M KCl, 5 mM indomethacin, and 2 mM EGTA. The samples were then centrifuged at 6,000 × g for 3 min, and the supernatant was collected for the quantification of thromboxane B_2_ (TxB_2_) production. TXB_2_ levels were measured using a commercially available TXB_2_ generation kit (Enzo Life Sciences, Farmingdale, NY) according to the manufacturer’s protocol.

### 2.8 Flow cytometry analysis

P-selectin and αIIbβ3, established biomarkers of platelet activation, were quantified via flow cytometry (Gallios, Beckman Coulter, Brea, CA, United States). Mouse platelets were pre-incubated with varying concentrations of ETN102 (100, 300, and 500 µM) or vehicle control (distilled water) for 15 min at 37 °C. Following this, platelets were activated with thrombin for 5 min at 37 °C to induce platelet activation. The activation status was subsequently assessed using antibodies targeting P-selectin and αIIbβ3.

### 2.9 Cytotoxicity measurement

Washed mouse platelets (3 × 10^8^ cells/mL) were incubated with vehicle or ETN102 (100, 300, and 500 µM) for 15 min at 37 °C and centrifuged at 900 × g for 8 min. LDH activity in the supernatant was measured using an LDH cytotoxicity assay kit (Abcam, Cambridge, United Kindom) according to the manufacturer’s instructions. Platelets treated with 1% Triton X-100 were used as the maximum LDH-release control. HT22 cells were seeded in 96-well plates at 3,000 cells/well and treated with vehicle or ETN102 (100, 300, and 500 µM) for 24 h. Cell viability was measured using a CCK-8 assay kit (Dojindo Laboratories, Kumamoto, Japan) and expressed as a percentage of the vehicle control. Platelet LDH release and HT22 cell viability were expressed relative to the maximum-release and vehicle controls, respectively.

### 2.10 Ca^2+^ mobilization

Mouse platelets (1 × 10^8^ cells/mL) were suspended in HEPES-Tyrode buffer without CaCl_2_. The platelets were pre-treated with varying concentrations of ETN102 (100, 300, and 500 µM) or vehicle control (distilled water) for 10 min at 37 °C. Subsequently, the platelets were stained with a Ca^2+^ indicator dye (FLIPR Ca^2+^ 5 analysis kit) in the dark for 30 min at 37 °C. Platelet activation was induced using 0.05 U/mL thrombin. Intracellular Ca^2+^ levels were quantified using a spectrophotofluorometer (Spectramax I3, Molecular Devices) at an excitation wavelength of 505 nm.

### 2.11 Immunoblotting

Mouse platelets were stimulated with thrombin (0.05 U/mL) and agitated at 1,000 rpm using an aggregometer to assess the phosphorylation levels of kinases associated with ETN102 at concentrations of 100, 300, and 500 μM. Platelets (6 × 10^8^ platelets/mL) were subsequently lysed in an equal volume of ice-cold 2 × lysis buffer (TBS, pH 7.4) containing 2% Triton X-100, 0.1% SDS, 2 mM EDTA, 2 mM Na_3_VO_4_, phosphatase inhibitor cocktail, protease inhibitor cocktail, and 2 mM phenylmethylsulfonyl fluoride. The lysates were centrifuged at 12,000 rpm for 15 min to separate the supernatant. Protein samples (30 μg) were subjected to electrophoresis under reducing conditions and immunoblotting, followed by reprobing with various antibodies. The expression of indicated proteins was detected using a digital imaging system (Q9 Alliance, UVITEC Ltd., Cambridge, United Kingdom). Band densities were quantified by densitometry using ImageJ software (v1.52a). Phosphorylation levels of kinases were determined by normalizing the density of phosphorylated kinases to that of total kinases.

### 2.12 Platelet spreading assay

Adhesive slides were coated with human fibrinogen at a concentration of 100 μg/mL and incubated overnight at 4 °C. Mouse platelets were pre-treated with ETN102 at concentrations of 100, 300, and 500 μM, or with vehicle control (distilled water), for 10 min at 37 °C. Subsequently, platelets (2 × 10^7^/mL) were allowed to adhere to and spread on the fibrinogen-coated wells for 2 h at 37 °C following thrombin stimulation. After washing, the cells were fixed with 3% paraformaldehyde, permeabilized using 0.1% Triton X-100, blocked with 0.1% BSA, and stained with 0.1 μg/mL rhodamine-conjugated phalloidin (Sigma). Images were acquired using an Olympus IX73 microscope (Seocho, Seoul, Korea) equipped with a 100x/1.3 NA oil immersion objective lens and captured with an Andor Zyla sCMOS camera. Imaging parameters for each fluorochrome were maintained consistently across all experimental conditions using MetaVue software (version 7.8.3.0). Adherent and spreading platelets were quantified within an area of 0.006 mm^2^ across five randomly selected fields. The surface area of spread platelets was measured in pixels using ImageJ software (v1.52a).

### 2.13 Clot retraction assay

Mouse blood was collected using 0.38% sodium citrate as an anticoagulant, followed by the isolation of platelets employing a standard protocol. The isolated platelets (5 × 10^7^ cells/mL) were subsequently combined with mouse platelet-poor plasma and treated with varying concentrations of ETN102 (100, 300, and 500 μM). Clot retraction was initiated by the addition of 0.2 U/mL thrombin and 250 μg/mL fibrinogen at 37 °C. After a 2-h incubation period, clot formation was documented photographically, and the clot volume for each treatment group was quantified using ImageJ software (v1.52a) for graphical analysis.

### 2.14 FeCl_3_-induced *in vivo* thrombosis

Mice were administered ETN102 intravenously via the left jugular vein at doses of 40 mg/kg body weight (BW) or 83 mg/kg BW. Additionally, ASA at a dose of 100 mg/kg BW was administered orally 4 h before the experiment. After 20 min, the right carotid artery was surgically exposed, and a filter paper (2 mm in diameter) saturated with 10% (460 mM) ferric chloride (FeCl_3_) was applied to the artery for 2 min. Subsequently, blood flow was monitored for up to 10 min post-occlusion using a blood flowmeter (AD Instruments, Dunedin, New Zealand).

### 2.15 Tail bleeding time

Mice were anesthetized with 2% isoflurane in an oxygen mixture, followed by intravenous administration of ETN102 at doses of 40 or 83 mg/kg BW via the left jugular vein, or ASA at 100 mg/kg BW was administered orally 4 h before the experiment. After 20 min, a 5 mm section of the tail was excised using a razor blade, and the tail was immersed in 13 mL of PBS prewarmed to 37 °C within a 15 mL tube. Tail bleeding was monitored, and the time to cessation of blood flow was recorded while maintaining the body temperature at 37 °C using a heating pad.

### 2.16 Middle cerebral artery occlusion (MCAO) surgery

Mice were anesthetized with isoflurane (induction at 2%, maintenance at 1%), and the left cervical region was shaved and disinfected. A cervical incision was performed to expose the common carotid artery (CCA), internal carotid artery (ICA), and external carotid artery (ECA). The CCA and ICA were ligated using sutures, and a silicone-coated 7-0 monofilament (L.M.S. Korea Inc., Gyeonggi, Korea) was inserted through the CCA and advanced to occlude the middle cerebral artery (MCA), thereby obstructing blood flow. Throughout the procedure, mice were maintained at a body temperature of 37 °C using a heating pad. After 50 min, the filament was withdrawn, and the surgical site was ligated and disinfected. Mice were monitored until full recovery of consciousness. ETN102 (40, 83 mg/kg, BW) was delivered via the internal jugular vein, or ASA at 100 mg/kg BW was administered orally 4 h before the experiment. Following 24 h of reperfusion, mice were euthanized, and their brains were harvested. Coronal brain slices of 2 mm thickness were stained with 2% 2,3,5-triphenyltetrazolium chloride (TTC) solution for 10 min at room temperature, followed by fixation in 4% paraformaldehyde. Infarct volume was quantified as the percentage of total non-stained volume (white) area relative to the total brain volume using ImageJ software (v1.52a).

### 2.17 Statistical analysis

Data were analyzed using GraphPad Prism 5 and are presented as mean ± SD or as individual values with the mean, as indicated. Multiple-group comparisons were performed using one-way ANOVA followed by Dunnett’s or Tukey’s multiple-comparisons test, as indicated, whereas two-group comparisons were performed using Student’s *t*-test. Occlusion time, bleeding time, and hemoglobin content were analyzed using one-way ANOVA followed by Tukey’s multiple-comparisons test. All reported n values represent biological replicates. For the *in vitro* platelet experiments, each biological replicate represents an independent platelet preparation obtained from an individual mouse, whereas n represents individual animals in the *in vivo* experiments. Sample sizes are provided in the corresponding figure legends, and P < 0.05 was considered statistically significant.

## 3 Results

### 3.1 ETN102 selectively inhibits thrombin-induced platelet aggregation

We initially conducted platelet aggregation assays to evaluate the effect of ETN102 on platelet function. Mouse platelets were pretreated with ETN102 at concentrations of 100, 300, or 500 μM, or with vehicle (distilled water), followed by stimulation with thrombin (0.05 U/mL). Our results demonstrated that ETN102 significantly inhibited thrombin-induced platelet aggregation compared to the vehicle control group. This inhibitory effect was consistent across all tested concentrations (Figure 2A). In contrast, ETN102 did not significantly inhibit platelet aggregation induced by intermediate concentrations of collagen (≤1 μg/mL), ADP (≤10 μM), or U46619 (≤1 μM) (Supplementary Figure S1). These findings indicate that ETN102 selectively inhibits thrombin-mediated platelet aggregation without affecting other agonist responses. Furthermore, we assessed ATP release and thromboxane B_2_ (TXB_2_) generation as indicators of ADP-dependent secondary amplification and TXA_2_ production, respectively, during platelet aggregation (Woulfe et al., 2001; Rivera et al., 2009). ETN102 significantly reduced ATP release during thrombin-induced aggregation (Figure 2B), whereas TXB_2_ generation remained unchanged (Supplementary Figure S2). These results suggest that ETN102 exerts its antiplatelet effect through a mechanism distinct from thromboxane-mediated signaling. To further clarify the basis of reduced ATP release, platelets were treated with 2 mM ethylene glycol-bis(β-aminoethyl ether)-N, N, N′, N′-tetraacetic acid (EGTA) to prevent the interaction between fibrinogen (FG) and activated αIIbβ3 integrin. Under these non-aggregating conditions, ETN102 no longer reduced ATP secretion (Figure 2C). Because ATP release in the presence of EGTA reflects primary agonist-driven secretion rather than integrin-dependent secondary amplification (Kim et al., 2013), these data suggest that ETN102 mainly suppresses ATP release indirectly by attenuating platelet-platelet aggregation and integrin-dependent reinforcement rather than by directly inhibiting thrombin-induced dense granule secretion. ETN102 did not significantly increase LDH release from washed platelets at concentrations up to 500 μM (Supplementary Figure S5A) or reduce HT22 cell viability following 24-h exposure at the tested concentrations (Supplementary Figure S5B). These findings indicate that ETN102 did not induce detectable platelet membrane damage or neuronal cytotoxicity under the experimental conditions used.

**FIGURE 2:**
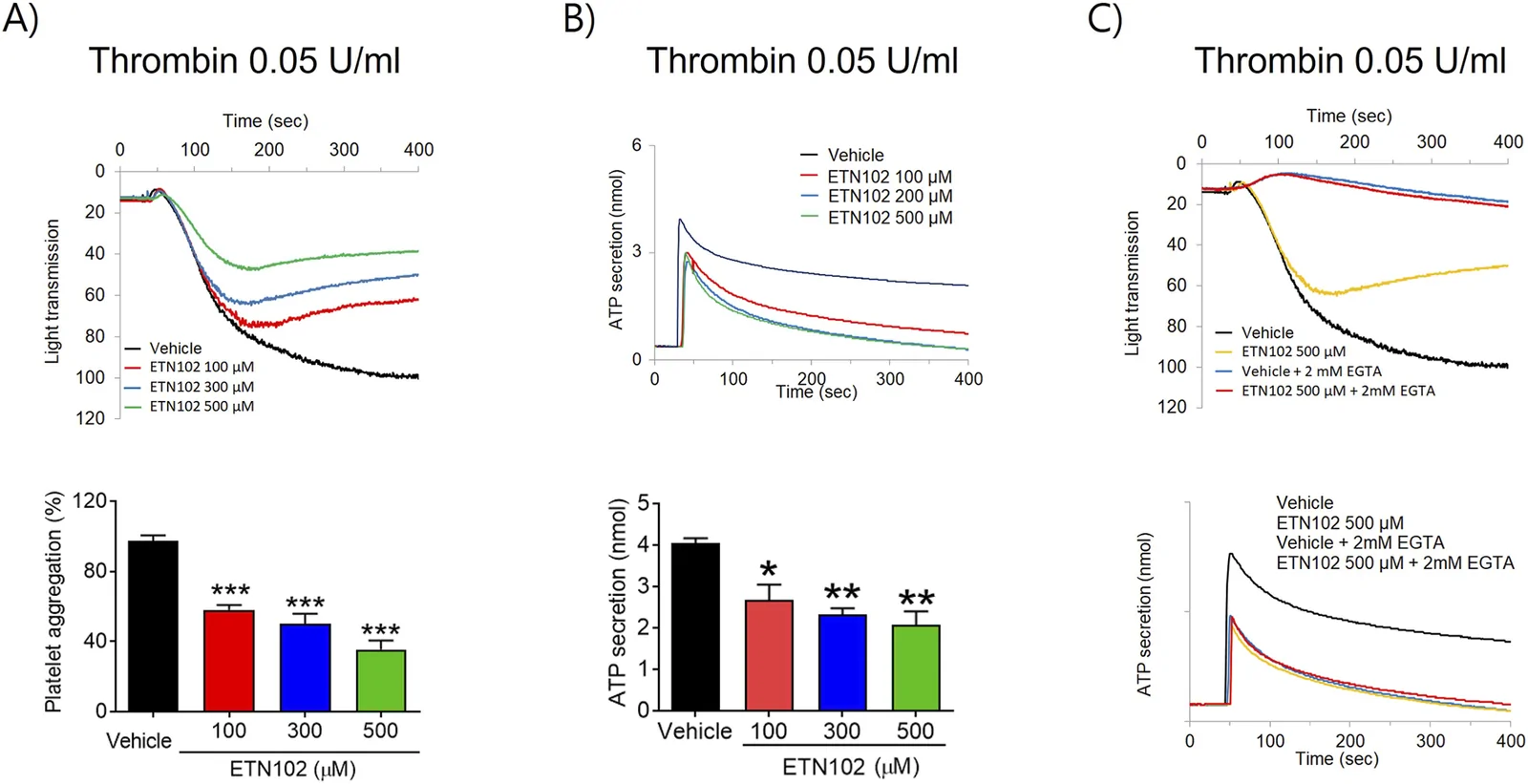
Inhibitory effect of ETN102 on thrombin-induced platelet aggregation and ATP secretion. Washed platelets were preincubated with ETN102 (100, 300, or 500 µM) for 15 min at 37 °C and subsequently stimulated with thrombin (0.05 U/mL). Platelet aggregation was measured under constant stirring conditions (1,000 rpm) using a Chrono-Log aggregometer **(A)**. For ATP secretion assays, platelets were preincubated with the same concentrations of ETN102 for 10 min at 37 °C, then luciferin/luciferase reagent was added before thrombin stimulation (0.05 U/mL). Luminescence was recorded to quantify ATP release **(B)**. In some experiments, mouse platelets were preincubated with a high concentration of ETN102 (500 µM) and activated with thrombin (0.05 U/mL) in the presence of EGTA (2 mM). **(C)** Aggregation and ATP secretion were monitored using a platelet aggregometer. Data are presented as the mean ± SD (n = 5). Statistical comparison with vehicle controls was performed using ANOVA followed by Dunnett’s test. Significance levels: *P < 0.05, **P < 0.01, and ***P < 0.001.

### 3.2 Treatment with ETN102 influences αIIbβ3 integrin activation but does not affect α-granule secretion and calcium mobilization

Next, we sought to examine the effect of ETN102 on platelet activation. The effects of ETN102 on αIIbβ3 integrin activation and P-selectin surface exposure, representing integrin activation and α-granule secretion, respectively, were assessed using flow cytometry (Bergmeier et al., 2002). Consistent with earlier findings, selective inhibition of platelet aggregation was observed under thrombin stimulation. Therefore, the present study specifically investigated the inhibitory effects of ETN102 on thrombin-induced platelet activation. Our findings demonstrated that ETN102 significantly inhibited thrombin-induced αIIbβ3 integrin activation in a dose-dependent manner (Figure 3A), whereas P-selectin exposure was unchanged (Figure 3B). These results indicate that ETN102 attenuates thrombin-induced αIIbβ3 integrin activation without significantly affecting α-granule secretion. Because intracellular Ca^2+^ mobilization is a major early event in platelet activation (Varga-Szabo et al., 2011), we examined whether ETN102 affected thrombin-induced Ca^2+^ signaling. Thrombin markedly increased both intracellular Ca^2+^ release and influx, but ETN102 had no significant effect on either Ca^2+^ release (Supplementary Figure S3A) or influx (Supplementary Figure S3B). Collectively, these data suggest that ETN102 selectively inhibits αIIbβ3 integrin activation, while not affecting α-granule secretion or intracellular Ca^2+^ mobilization.

**FIGURE 3:**
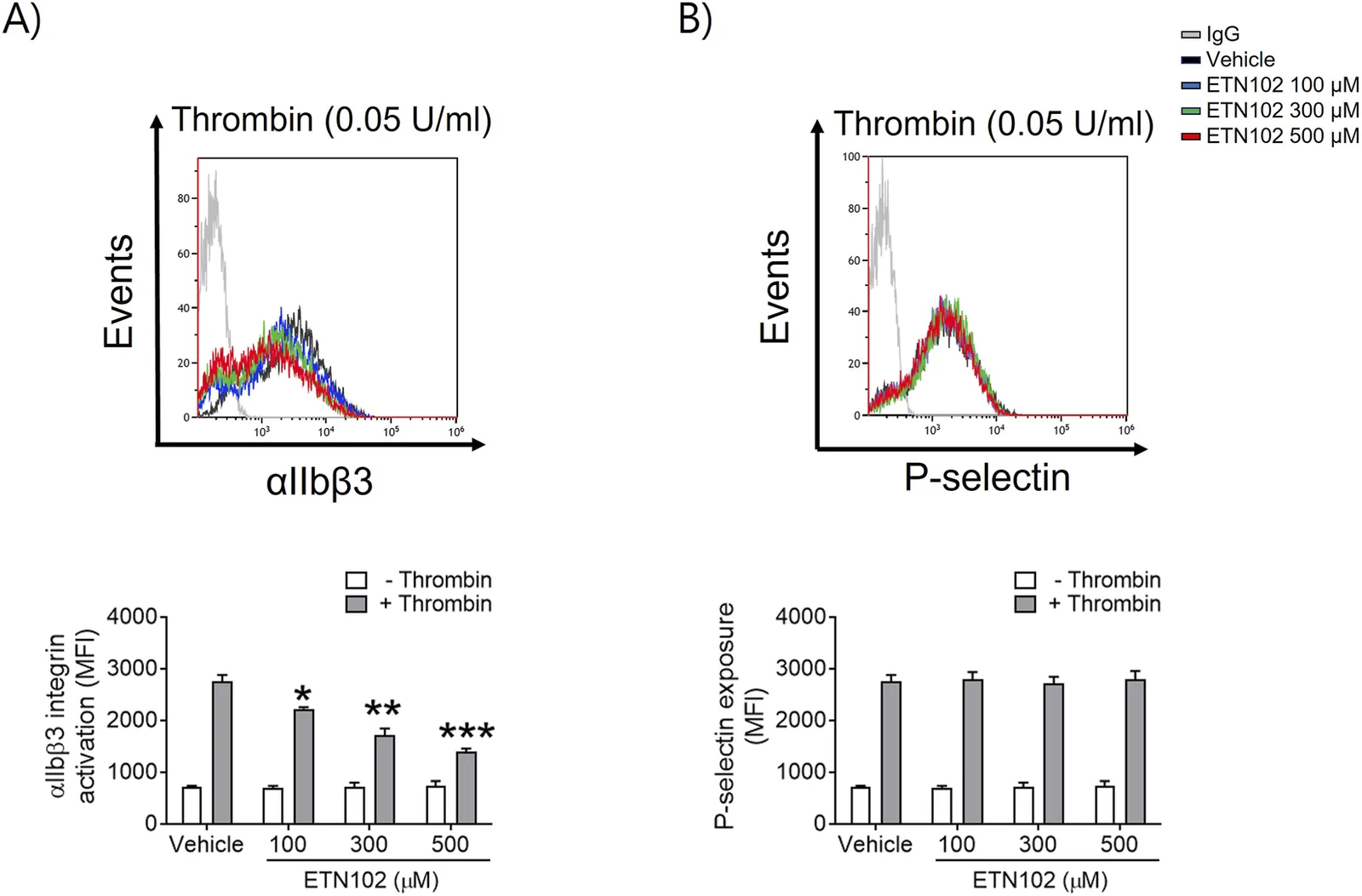
ETN102 regulates αIIbβ3 integrin activation without affecting P-selectin exposure during cell activation. Flow cytometry was used to assess the effects of ETN102 on thrombin-induced αIIbβ3 integrin activation and P-selectin exposure. Washed platelets were preincubated with ETN102 (100, 300, or 500 µM) for 15 min at 37 °C and subsequently stimulated with thrombin (0.05 U/mL). Binding of anti-activated αIIbβ3 (JON/A) antibodies **(A)** and anti-P-selectin antibodies **(B)** was quantified by calculating the ratio of geometric mean fluorescence intensity (MFI) relative to isotype controls. Data represent mean ± SD (n = 5). Statistical analyses were performed using ANOVA followed by Dunnett’s test. Significance levels: *P < 0.05, **P < 0.01, and ***P < 0.001 compared to the vehicle control.

### 3.3 Effects of ETN102 on platelet spreading and clot retraction

Activated integrin αIIbβ3 binds to fibrinogen and triggers a cascade of outside-in signaling events within platelets, culminating in platelet spreading and clot retraction (Durrant et al., 2017). Considering that ETN102 treatment modulates platelet aggregation and ATP secretion via regulation of αIIbβ3 integrin activation, we further investigated its impact on outside-in signaling by employing platelet spreading and clot retraction assays. Treatment with ETN102 induced a concentration-dependent inhibition of platelet spreading on fibrinogen (Figures 4A, B) and significantly reduced clot retraction (Figures 4C, D) in the presence of thrombin, compared to the vehicle control. Notably, among all examined concentrations, 500 µM ETN102 exhibited the most substantial inhibitory effects on both platelet spreading and clot retraction. These findings suggest that ETN102 disrupts αIIbβ3 integrin-mediated outside-in signaling, potentially through modulation of its ligand-binding activity.

**FIGURE 4:**
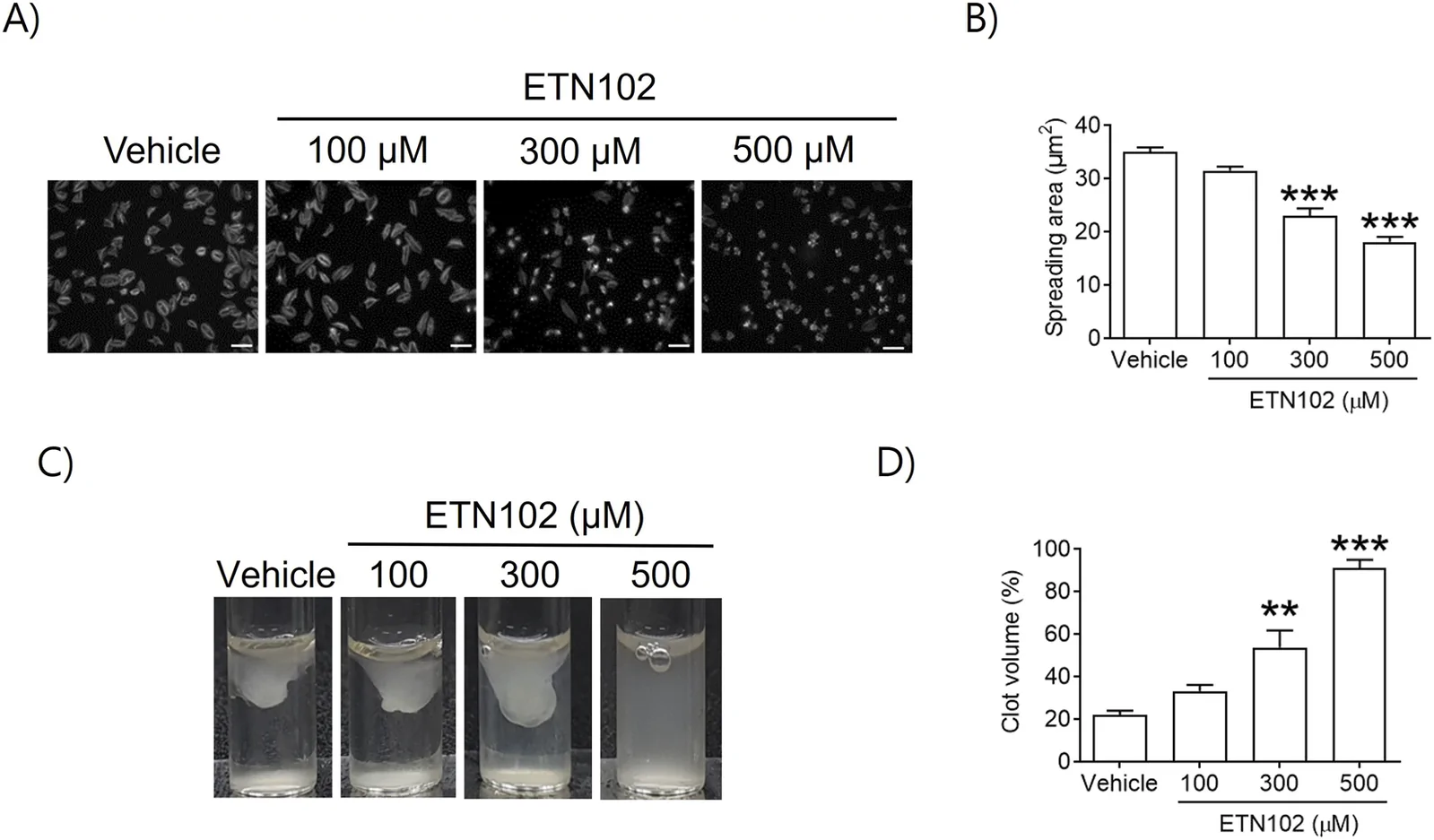
ETN102 impairs αIIbβ3 integrin-dependent platelet spreading and clot retraction. ETN102 was evaluated for its effects on platelet spreading on immobilized fibrinogen and clot retraction induced by thrombin. Washed platelets were pretreated with ETN102 (100, 300, or 500 µM) or a vehicle control. **(A, B)** Platelets were allowed to adhere and spread on fibrinogen-coated wells following thrombin stimulation. **(C, D)** Clot retraction was assessed up to 2 h after adding thrombin, fibrinogen, and CaCl_2,_ with representative photographs **(C)** and corresponding summary data **(D)** presented. Data are expressed as mean ± SD (n = 5). Statistical significance was determined by ANOVA followed by Dunnett’s test. **P < 0.01, and ***P < 0.001 compared with vehicle control.

### 3.4 ETN102 plays a pivotal role in modulating αIIbβ3 integrin outside-in signaling

The activation of the αIIbβ3 integrin outside-in signaling pathway induces the phosphorylation of key downstream signaling molecules, particularly focal adhesion kinase (FAK) and proline-rich tyrosine kinase 2 (Pyk2). These kinases play central roles in mediating cytoskeletal reorganization and mechanical force transmission, thereby promoting platelet spreading, clot retraction, and stabilizing thrombus formation (Guan, 1997; Cipolla et al., 2013; Durrant et al., 2017; Guidetti et al., 2019). Accordingly, we evaluated the phosphorylation status of these signaling intermediates. Initially, we investigated the selective suppression of COX-2 expression by ETN102 using Western blot analysis, given its known selective inhibitory effect on COX-2 (Kim et al., 2020). The results demonstrated a decrease in COX-2 expression following ETN102 treatment, whereas COX-1 levels remained unchanged (Figures 5A, B). Because platelet-derived thromboxane synthesis is driven predominantly by COX-1, the preserved COX-1 expression is consistent with the absence of a significant change in TXB_2_ generation. Consistent with the observed impairment in platelet clot retraction, platelets treated with ETN102 exhibited significantly reduced phosphorylation of FAK and Pyk2 upon thrombin stimulation compared to vehicle control (Figures 5C, D). In contrast, no alterations were detected in the phosphorylation of Syk, PI3K, and AKT following stimulation (Supplementary Figure S4). These results indicate that the reduction in phosphorylation of FAK and Pyk2 under integrin-engaging conditions is consistent with impaired outside-in signaling, potentially associated with reduced platelet-platelet aggregation. Conversely, ETN102 did not influence intrinsic platelet activation pathways, as evidenced by the unchanged phosphorylation levels of Syk, PI3K, and AKT. Collectively, these findings suggest that ETN102 does not suppress agonist-induced platelet activation signaling but selectively attenuates thrombin-context αIIbβ3 integrin-mediated outside-in signaling.

**FIGURE 5:**
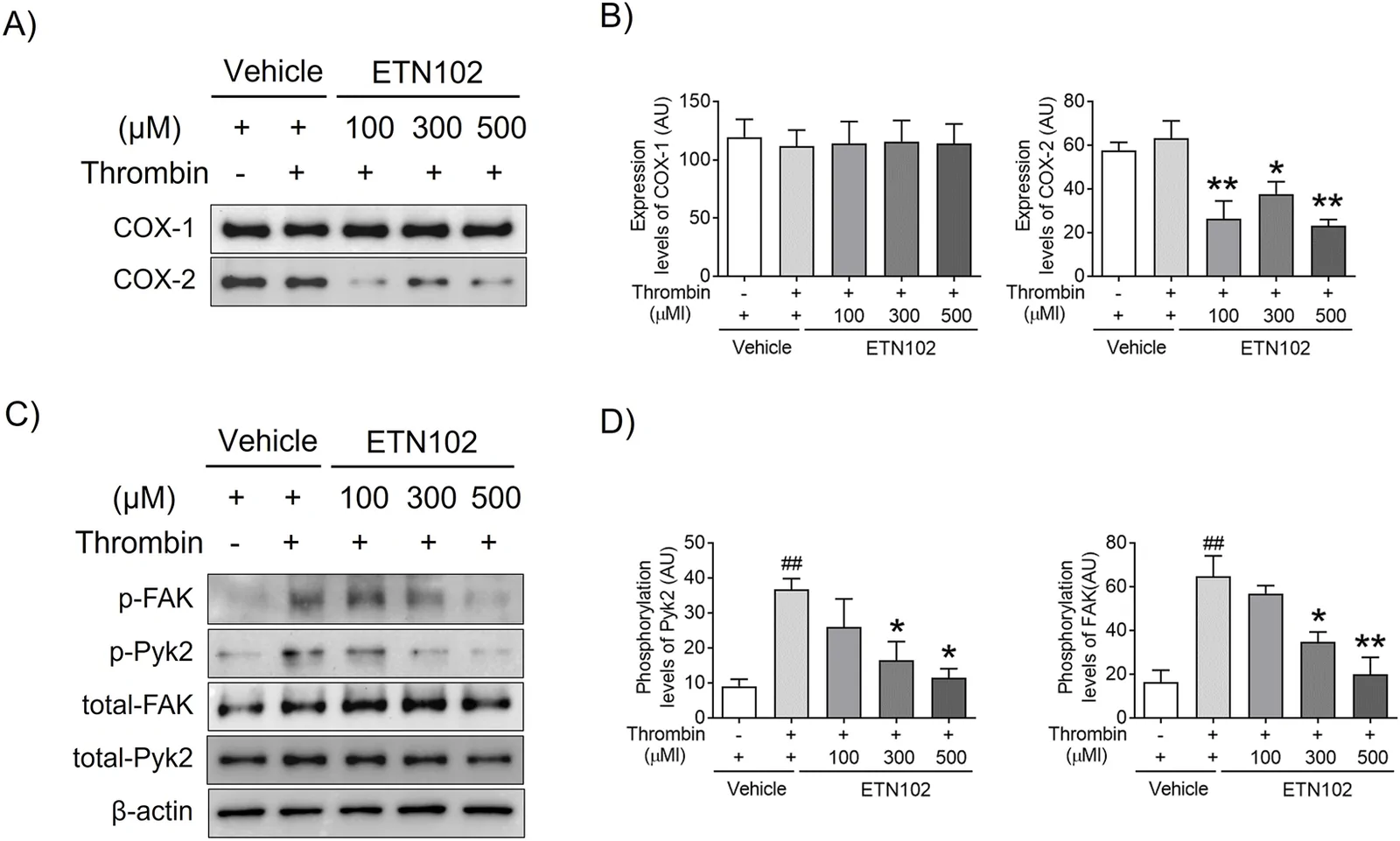
ETN102 selectively suppresses COX-2 expression and FAK/Pyk2 phosphorylation without affecting COX-1 in thrombin-stimulated platelets. Mouse platelets were pre-treated with ETN102 (100, 300, or 500 µM) and stimulated with 0.05 U/mL Thrombin. **(A)** Representative immunoblots showing the expression levels of COX-1 and COX2. **(B)** Quantitative analysis of COX-1 and COX-2 expression levels. **(C)** Representative immunoblots showing phosphorylation levels of FAK and Pyk2. **(D)** Quantitative analysis of phosphorylated FAK and Pyk2 levels. Data are presented as mean ± SD (n = 5). ##P < 0.01 versus the vehicle control (unstimulated) after Student’s *t*-test, and *P < 0.05 and **P < 0.01 versus vehicle control (stimulated) after ANOVA and Dunnett’s test.

### 3.5 ETN102 inhibits thrombosis *in vivo* within a murine model of carotid artery thrombosis

To evaluate the effects of ETN102 on arterial thrombus formation, a FeCl_3_-induced carotid artery thrombosis model was utilized in mice. Anesthesia was induced with 2% isoflurane, and ETN102 was administered intravenously via the left jugular vein at doses of 40 or 83 mg/kg BW. ASA was administered orally at a dose of 100 mg/kg BW and served as a positive control. Twenty minutes post-administration, the right carotid artery was exposed, and a 2 mm diameter filter paper saturated with 10% FeCl_3_ was applied to the artery for 2 min to induce thrombosis. In the vehicle-treated control group, carotid artery occlusion occurred at a mean time of 12.2 ± 1.2 min following FeCl_3_ application. In contrast, the mean time to blood flow occlusion was significantly prolonged in the low-dose ETN102 group (29.4 ± 0.3 min) and the high-dose ETN102 group (38.2 ± 0.8 min). Notably, ETN102 administration resulted in occlusion times comparable to those observed with ASA treatment (33.2 ± 0.3 min) (Figures 6A, B). These results demonstrate that ETN102 exerts antithrombotic activity *in vivo*, consistent with its inhibitory effects on platelet aggregation and thrombus stabilization.

**FIGURE 6:**
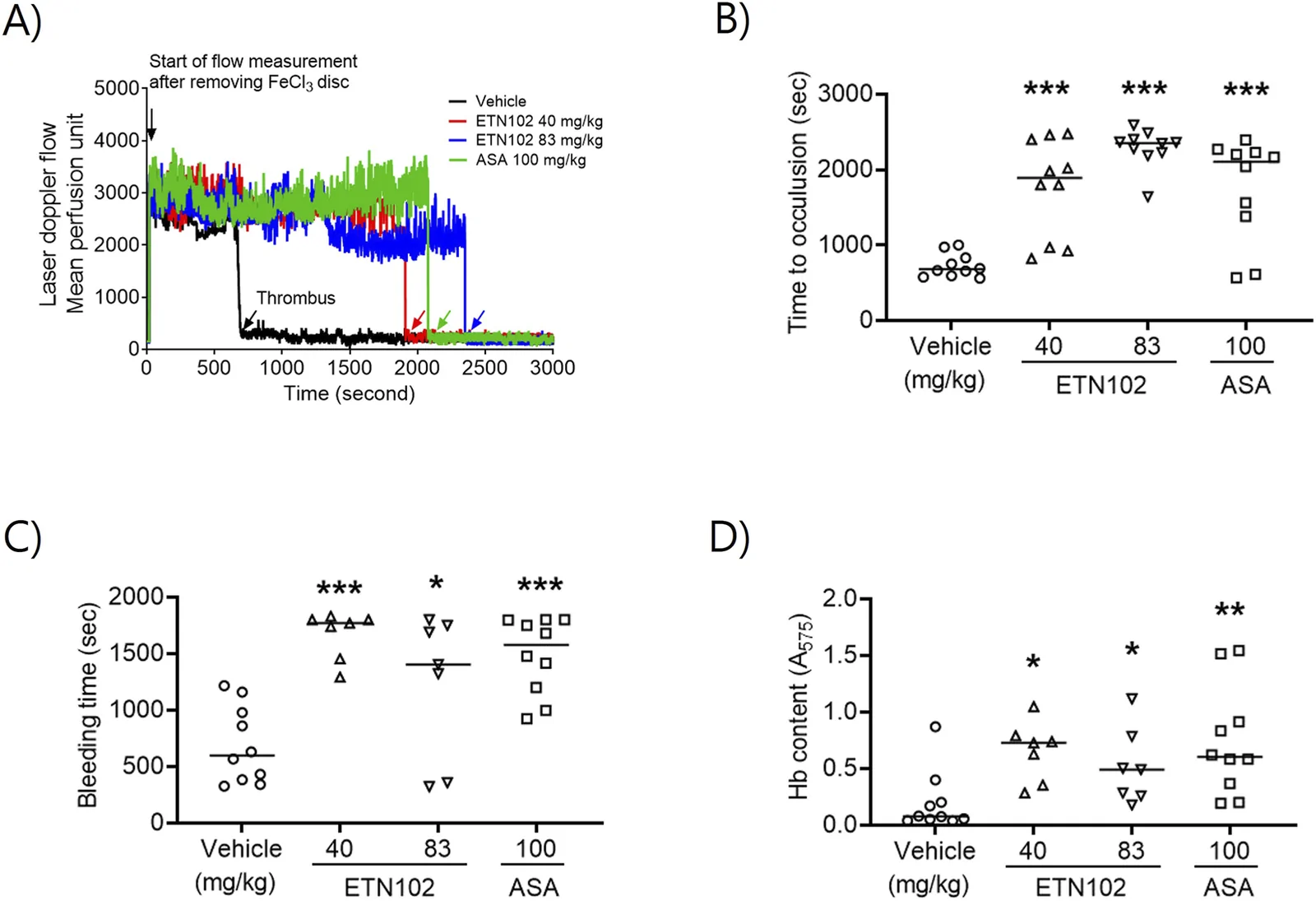
Role of ETN102 in thrombus formation and tail bleeding time. The FeCl_3_-induced arterial thrombus formation was carried out according to the methods described. Following the intravenous administration of ETN102 (40, 83 mg/kg BW) via the left jugular vein or oral administration of aspirin (ASA, 100 mg/kg BW) 4 h before the experiment, **(A)** the right carotid artery of the mouse was treated with 10% FeCl_3_ for 2 min. The blood flow traces were continuously monitored until a stable occlusion occurred. **(B)** Individual data points are shown, and horizontal bars indicate the mean (n = 10 mice per group). In the bleeding time assay, the tails of the mice treated with the vehicle (circle), a low dose of ETN102 (40 mg/kg, triangle), a high dose of ETN102 (83 mg/kg, reverse triangle), and ASA (square) were amputated. **(C)** The bleeding time and **(D)** Hb content were monitored as described in the methods. Individual data points are shown, and horizontal bars indicate the mean (vehicle, n = 10; ETN102 40 mg/kg, n = 7; ETN102 83 mg/kg, n = 7; ASA, n = 10). Statistical comparisons for panels B–D were performed using one-way ANOVA followed by Tukey’s multiple-comparisons test. Significance is indicated as follows: *P < 0.05, **P < 0.01, and ***P < 0.001 versus the vehicle control.

### 3.6 Hemostatic effects of ETN102 in a tail bleeding time animal model

We further examined the impact of ETN102 administration at doses of 40 and 83 mg/kg BW on hemostatic function. Tail bleeding time was determined by measuring the duration until bleeding cessation following tail amputation. The results demonstrated that both ETN102 and the positive control, ASA, significantly prolonged tail bleeding time compared to the control group (Figure 6C). Additionally, quantification of blood loss at the amputation site, assessed via hemoglobin content, revealed a statistically significant increase in blood loss in the ETN102-treated groups relative to controls (Figure 6D), with effects comparable to those observed with ASA. These findings indicate that ETN102 administration significantly influences *in vivo* hemostasis.

### 3.7 ETN102 attenuates cerebral ischemia/reperfusion injury

We evaluated the effects of ETN102 using a murine model of brain injury induced by middle cerebral artery occlusion (MCAO) followed by reperfusion. MCAO resulted in cerebral infarction and neuronal damage. Notably, the infarct volume was significantly reduced from 31.58% in the control group to 21.69% and 16.48% in the ETN102-treated groups at 40 and 83 mg/kg BW, respectively, corresponding to reductions of approximately 31.3% and 47.8% compared to the control group (Figures 7A, B). Furthermore, we assessed the impact of ETN102 on neuronal damage and apoptosis after ischemic injury. Our results demonstrated that ETN102 treatment enhanced the expression of NeuN and Bcl-2 proteins (Figures 7C, D). These results suggest that ETN102 may contribute to both suppression of thrombus formation and attenuation of neuronal damage following ischemic injury.

**FIGURE 7:**
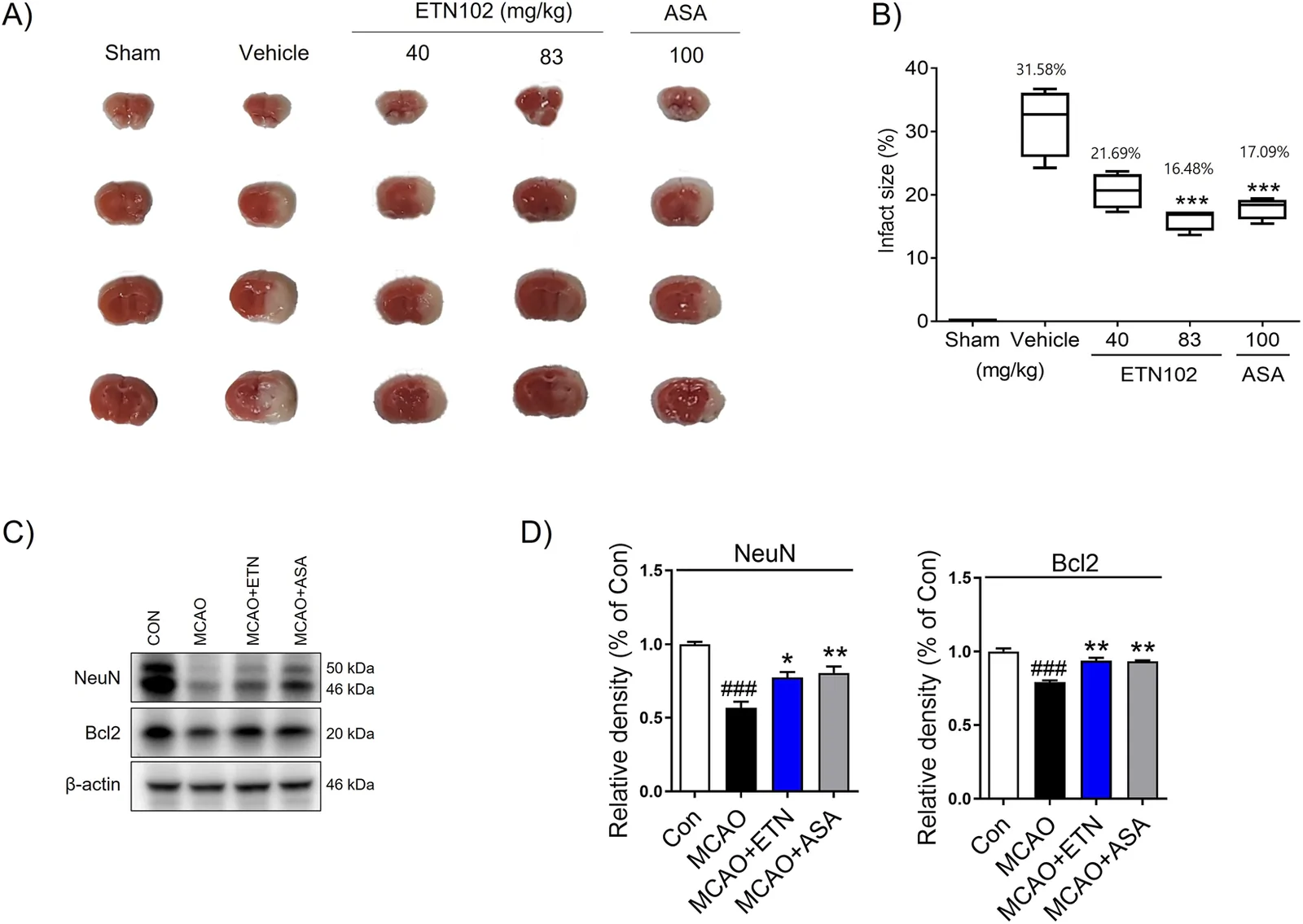
ETN102 plays a critical role in the pathogenesis of cerebral ischemia/reperfusion injury *in vivo.* The middle cerebral artery occlusion procedure and the detailed protocol are described in the Section 2. **(A)** Representative images of brain sections stained with 2,3,5-triphenyl tetrazolium chloride **(B)**. The infarct volume was measured by Image J. Data are presented as mean ± SD (n = 8 mice per group). Statistical significance is indicated as follows: ***P < 0.001 versus the vehicle control, determined by ANOVA followed by Tukey’s *post hoc* test. Neuronal damage and apoptosis were further evaluated by immunoblotting to assess NeuN and Bcl-2 protein expression. Representative blots are shown in panel **(C)**, and the corresponding densitometric analyses are presented in panel **(D)**. Data are expressed as mean ± SD (n = 5 mice per group). Statistical significance was determined using ANOVA and Tukey’s *post hoc* test: ###P < 0.001 versus the vehicle control, and *P < 0.05 and **P < 0.01 between the two groups.

## 4 Discussion

Nonsteroidal anti-inflammatory drugs (NSAIDs) exert their effects by inhibiting cyclooxygenase (COX), thereby suppressing the synthesis of prostaglandins responsible for inflammation, pain, and fever (Sohail et al., 2023; Ghlichloo and Gerriets, 2025). Diflunisal, a salicylic acid-derived NSAID, is traditionally classified as a nonselective COX inhibitor and is known to attenuate inflammatory responses through prostaglandin suppression (Carta et al., 2018; Snetkov et al., 2021). Recent studies have explored the conjugation of gadolinium with NSAIDs to develop therapeutic molecules that combine anti-inflammatory activity with enhanced magnetic resonance imaging (MRI) visualization of inflamed tissues (Sung et al., 2023; Ibrahim et al., 2025). Although gadolinium-based contrast agents (GBCAs) are primarily used for imaging, certain formulations have been suggested to influence cellular functions, including integrin-mediated signaling and calcium channel activity, which may modify adhesion or activation responses in specific cell types (Zhou and Lu, 2013; Ariyani et al., 2022). However, the role of such conjugated gadolinium-NSAID structures in platelet physiology remains poorly defined. In this study, we investigated the biological effects of ETN102, a gadolinium-conjugated diflunisal derivative, on platelet activation and thrombogenesis. ETN102 selectively inhibited thrombin-induced platelet aggregation and αIIbβ3 integrin activation while leaving collagen-, ADP-, and U46619-induced responses unaffected. It significantly reduced platelet spreading and clot retraction, key functions governed by αIIbβ3 outside-in signaling. These inhibitory effects were accompanied by a marked suppression of FeCl_3_-induced arterial thrombosis and altered hemostatic function in tail transection assays, indicating a physiological impact on thrombosis and hemostasis.

Despite its diflunisal backbone, ETN102 did not significantly reduce TXB_2_ generation, which was measured as the stable metabolite of TXA_2_. This finding is particularly important because it indicates that ETN102 does not act as a conventional platelet COX-1 inhibitor. This finding aligns with our observation that COX-1 protein expression remained unchanged, consistent with the well-established role of COX-1 as the dominant isoform responsible for platelet TXA_2_ synthesis (Faki and Er, 2021). In contrast, ETN102 selectively reduced COX-2 protein levels, a particularly interesting observation given that COX-2 in platelets is typically minimal according to prior reports (Rocca et al., 2002; LactMed, 2006; Hu et al., 2017; Ghlichloo and Gerriets, 2025). Therefore, the reduction in COX-2 expression is unlikely to account directly for the inhibitory effects of ETN102 on platelet aggregation. This effect may reflect an anti-inflammatory property of ETN102 that is more relevant in nucleated vascular or immune cells than in anucleate platelets. This observation suggests that gadolinium conjugation may alter the cellular uptake, protein-binding properties, or enzyme specificity of diflunisal, thereby conferring biological activities distinct from those of the parent compound. Since COX-2 is more involved in inflammatory prostaglandin production in nucleated stromal and immune cells, and selective COX-2 modulation generally has less impact on primary hemostasis compared with COX-1 inhibition (Kulkarni et al., 2000; Brainard et al., 2007), our findings suggest that the antiplatelet effect of ETN102 is largely independent of COX-1-mediated thromboxane pathways.

Mechanistically, the present data support a model in which ETN102 selectively modulates thrombin-dependent integrin-mediated platelet functional responses, rather than interfering with proximal agonist-induced activation signaling. ETN102 did not significantly alter phosphorylation of Syk, PI3K, or AKT, nor did it affect intracellular Ca^2+^ mobilization or P-selectin exposure, indicating that the compound does not broadly suppress classical intracellular activation pathways triggered by thrombin. Moreover, although αIIbβ3 outside-in signaling can involve Syk activity, our data showed preserved activation of Syk during integrin engagement, consistent with reports that Syk-deficient platelets can still undergo fibrinogen adhesion and thrombin-induced aggregation in certain contexts (Law et al., 1999; Obergfell et al., 2002). This finding supports the interpretation that ETN102 preferentially disrupts thrombin-associated integrin mechanical signaling events, rather than broadly suppressing canonical intracellular kinase cascades. Furthermore, it suggests that these effects may not be uniformly observed across platelet activation induced by other agonists. However, the present findings do not establish whether ETN102 directly binds to αIIbβ3, FAK, Pyk2, or another upstream regulatory molecule. ETN102 should therefore be considered a functional modulator of thrombin-associated αIIbβ3 outside-in signaling rather than a confirmed direct inhibitor of a specific molecular target. The thrombin-context selectivity of ETN102 defines both its potential utility and its limitations. ETN102 may preferentially interfere with integrin-dependent platelet aggregation and thrombus stabilization under conditions involving substantial thrombin generation, while providing limited inhibition of platelet responses driven predominantly by collagen, ADP, or thromboxane receptors. However, the present study did not directly compare ETN102 across disease-specific thrombotic settings or establish the relative contribution of thrombin signaling in the FeCl_3_-induced thrombosis and MCAO models. Therefore, the applicability of ETN102 to specific thrombotic conditions remains to be established. Moreover, the high concentrations required *in vitro* (100–500 μM), the intravenous doses used *in vivo* (40–83 mg/kg), and the observed prolongation of bleeding time indicate that further pharmacokinetic, dose-optimization, and safety studies are necessary before its clinical relevance can be determined. A schematic summary of the proposed mechanism by which ETN102 modulates thrombin-associated αIIbβ3 outside-in signaling and its functional consequences is presented in Figure 8.

**FIGURE 8:**
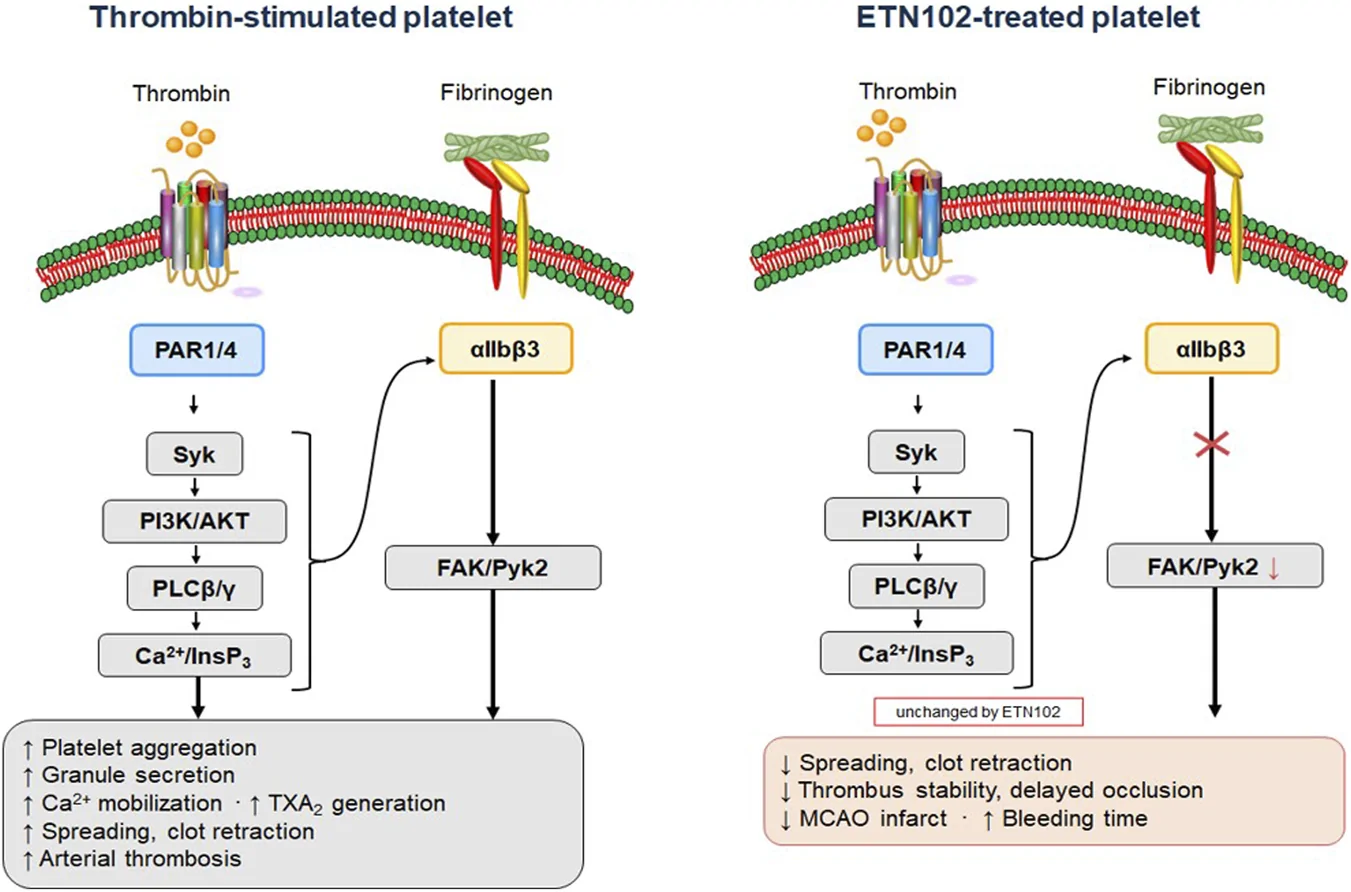
Proposed mechanism and functional consequences of ETN102-mediated platelet modulation. ETN102 selectively attenuates thrombin-context αIIbβ3 outside-in signaling, as supported by reduced FAK and Pyk2 phosphorylation, platelet spreading, clot retraction, and integrin-dependent secondary ATP release. ETN102 does not broadly suppress Ca^2+^ mobilization, P-selectin exposure, Syk/PI3K/AKT phosphorylation, or TXB_2_ generation, and it does not inhibit aggregation induced by collagen, ADP, or U46619 under the tested conditions. These platelet effects are associated with delayed FeCl_3_-induced arterial occlusion and prolonged bleeding *in vivo*. Reduced cerebral infarction and neuronal injury were also observed after MCAO.

Beyond their roles in hemostasis and thrombosis, platelets contribute to neurological diseases through thromboinflammatory signaling, interactions with leukocytes and the neurovascular unit, and the release of bioactive mediators (Rawish et al., 2020; Sun and Langer, 2022). Platelet functions may therefore influence neurovascular injury through mechanisms extending beyond vascular occlusion, although these effects are context-dependent and may include the regulation of vascular repair and tissue recovery (Rawish and Langer, 2022; Nording et al., 2023; Qiu et al., 2024). In the present study, ETN102 reduced infarct volume and preserved NeuN and Bcl-2 expression following MCAO/reperfusion injury. However, these findings do not distinguish whether the observed neuroprotection resulted from altered platelet-dependent thrombosis and cerebral perfusion, direct effects on neural or vascular cells, or the previously reported anti-inflammatory actions of ETN102 (Kim et al., 2020). Accordingly, the MCAO findings should be interpreted as an association between ETN102 treatment and reduced ischemic brain injury rather than definitive evidence that platelet inhibition directly mediated its neuroprotective effect.

ETN102 also demonstrated antithrombotic activity in FeCl_3_-induced arterial thrombosis models, with efficacy comparable to aspirin, a well-established antiplatelet agent whose clinical utility is often limited by bleeding complications (Altman et al., 2012; Godier et al., 2020). Notably, ETN102 produced comparable reductions in thrombus formation while simultaneously increasing bleeding risk, underscoring the need for rigorous dose refinement and comprehensive safety assessment before further development. Although ETN102 prolonged bleeding time under the present experimental conditions, its thrombin-context-selective mode of action remains mechanistically distinct from the COX-1-dependent activity of aspirin. Further, Dose-response and pharmacokinetic studies are needed to determine whether its antithrombotic activity can be optimized while minimizing its effects on hemostasis. In addition to these pharmacodynamic concerns, the presence of gadolinium warrants careful safety evaluation because gadolinium-based contrast agents have been associated with nephrotoxicity, tissue retention, and long-term safety risks, particularly in individuals with impaired renal function (Rogosnitzky and Branch, 2016; Rudnick et al., 2021; Iacobellis et al., 2024; Domingo and Semelka, 2025). Because conjugation to a pharmacologically active compound may alter biodistribution, stability, and clearance, future studies should characterize the pharmacokinetic and tissue-distribution profiles of ETN102 and evaluate its safety following single and repeated administration.

Overall, ETN102 is identified as a thrombin-context-selective modulator of platelet function, preferentially affecting αIIbβ3 outside-in signaling during thrombin stimulation, while sparing proximal platelet activation signaling and COX-1-dependent thromboxane synthesis. The concurrent inhibition of FAK/Pyk2 phosphorylation, platelet spreading, clot retraction, and arterial thrombus formation is consistent with modulation of integrin-dependent thrombus stabilization by ETN102. Together with its protective effects against cerebral ischemia/reperfusion injury, these findings support further investigation of the antithrombotic and neuroprotective properties of ETN102. Further studies are warranted to identify its direct molecular target, optimize the dose-response relationship, characterize its pharmacokinetic and safety profiles, and clarify the contribution of platelet modulation to its neuroprotective effects.

## Data Availability

The raw data supporting the conclusions of this article will be made available by the authors, without undue reservation.
