# Supplementary material for: The inhibitory effect of gadolinium-conjugated diflunisal on platelet function through regulating outside-in signaling of αIIbβ3 integrin
Source: Front Pharmacol. 2026 Oct 19;17:1936307. doi: 10.3389/fphar.2026.1936307 (PMC13725699; doi:10.3389/fphar.2026.1936307)
Supplement: Supplementary file 1 [file DataSheet1.pdf]

Appendix

COX-1(72kDa)

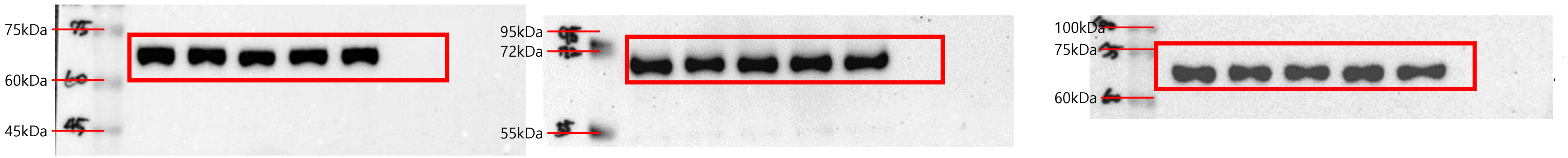

COX-2(72kDa)

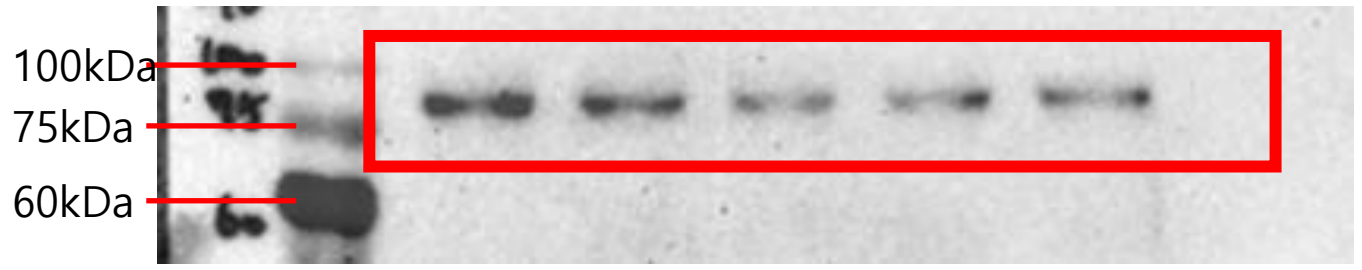

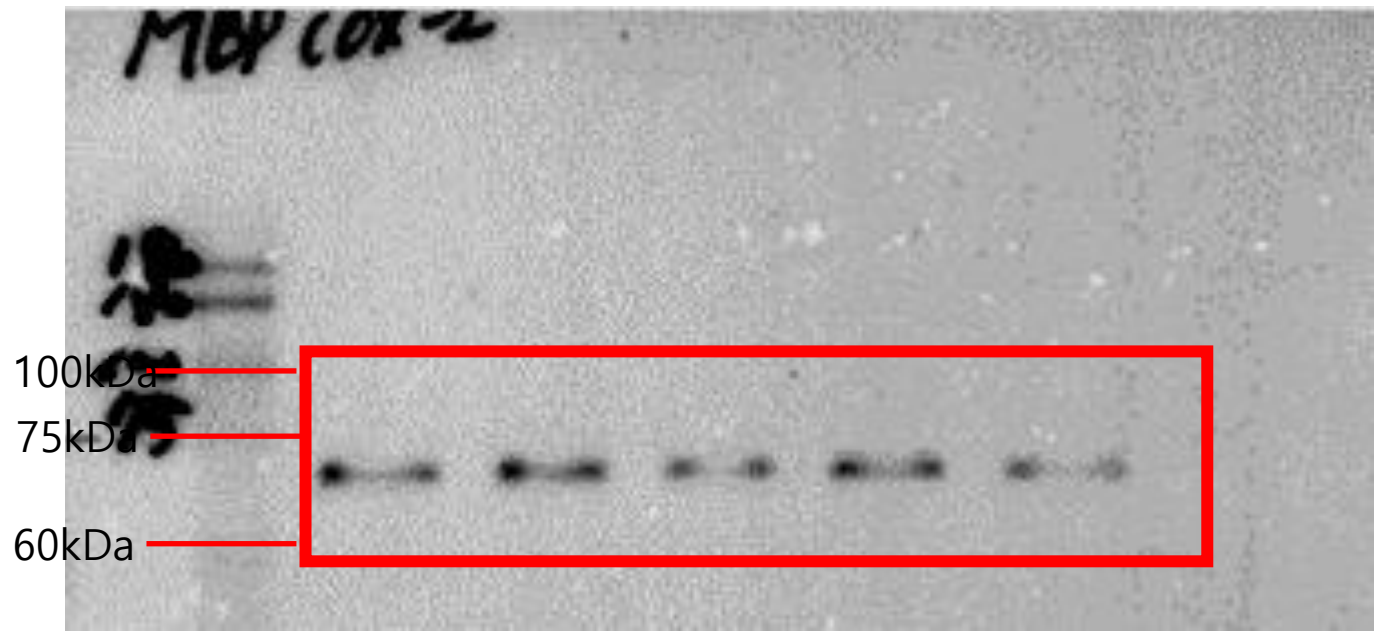

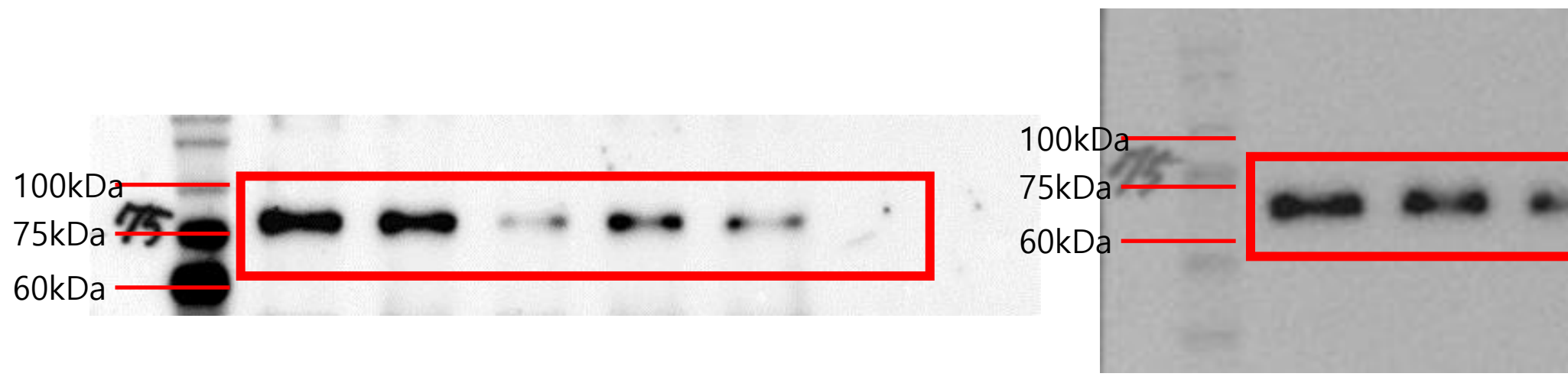

p-Src(60kDa)

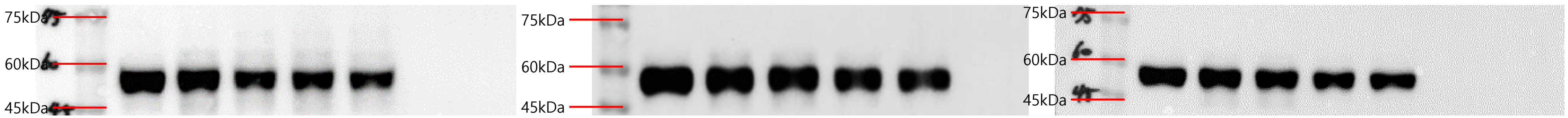

T-Src(60kDa)

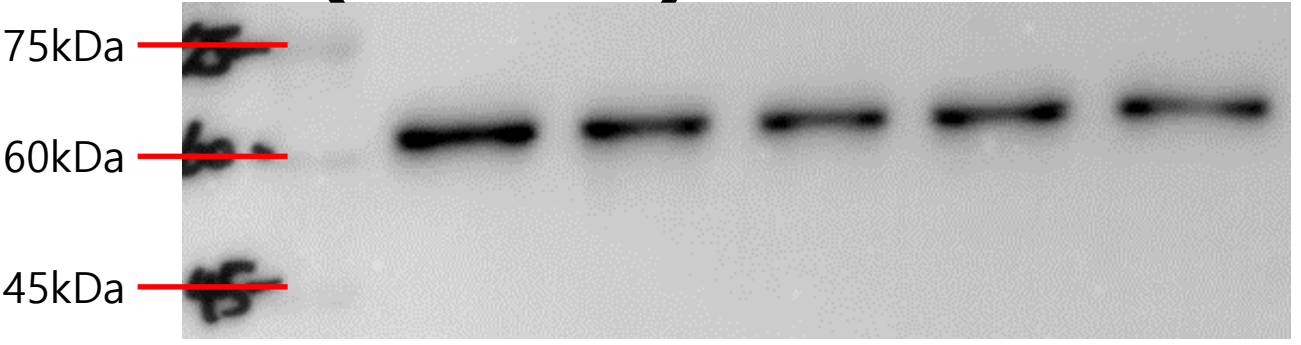

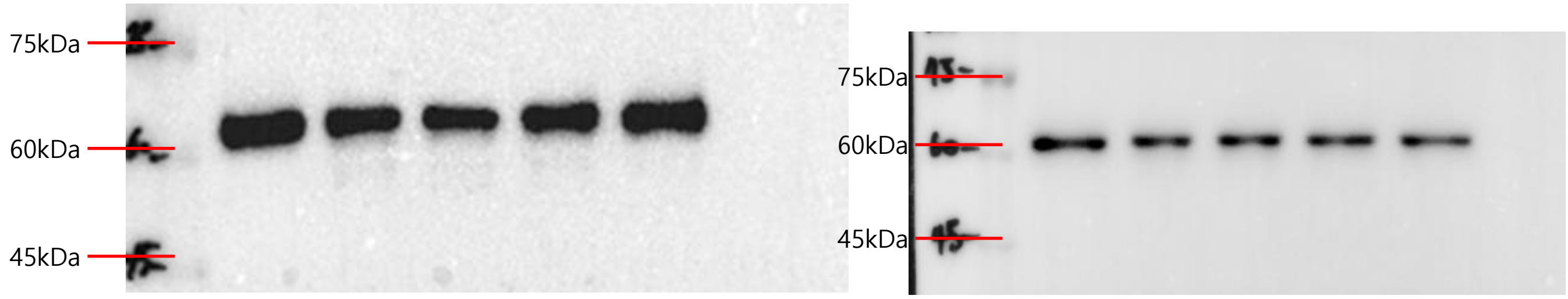

p-AKT(60kDa)

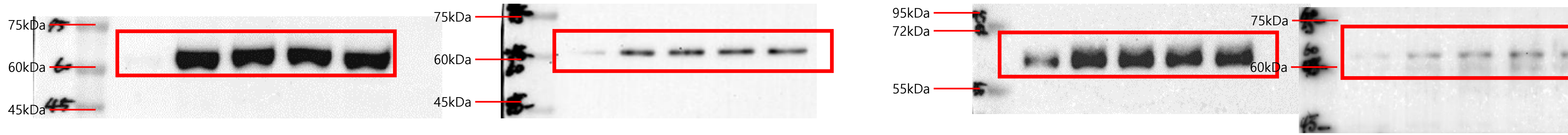

T-AKT(60kDa)

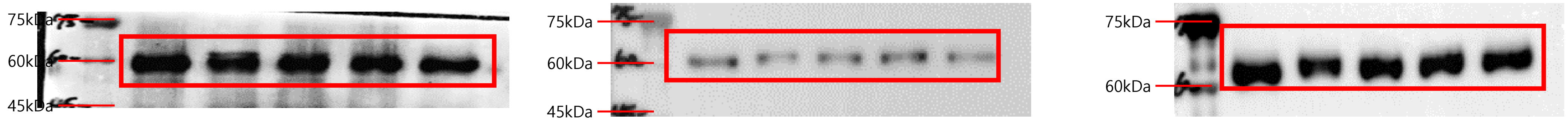

p-Syk(72kDa)

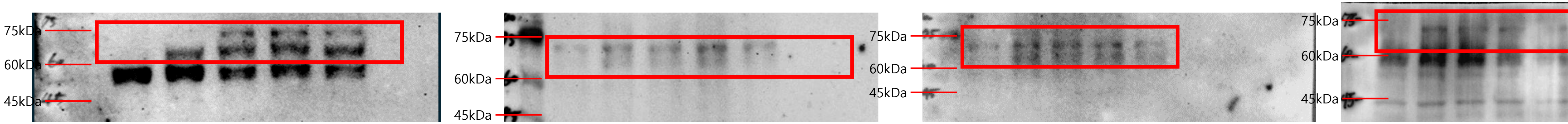

T-Syk(72kDa)

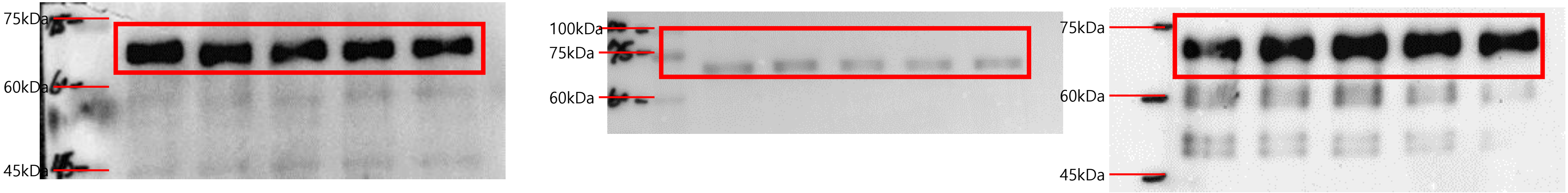

P-PI3K(60,85kDa)

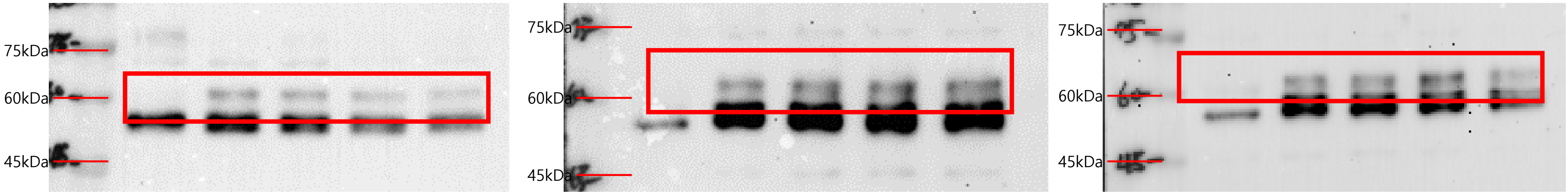

T-PI3K(110kDa)

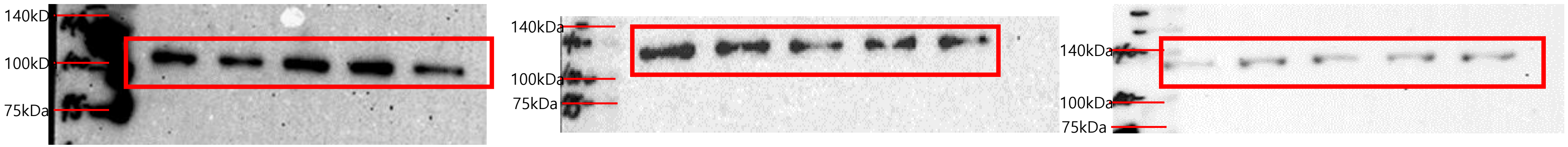

p-plcβ3(150kDa)

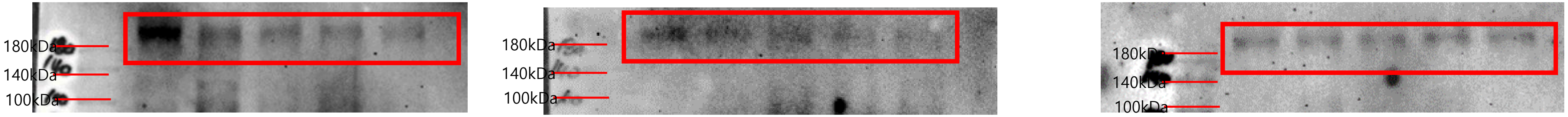

T-plcβ3(150kDa)

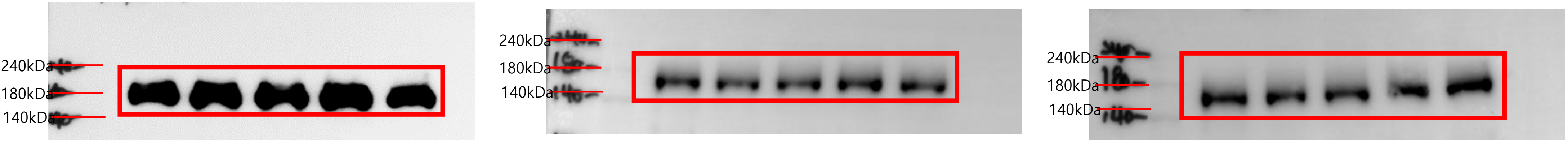

p-FAK(125kDa)

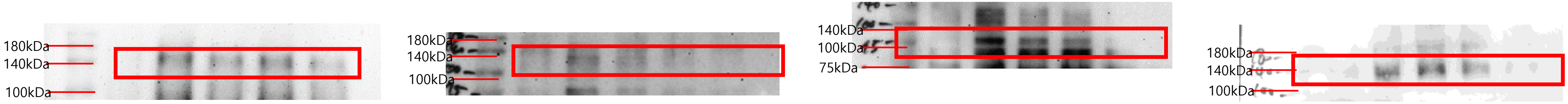

T-FAK(125kDa)

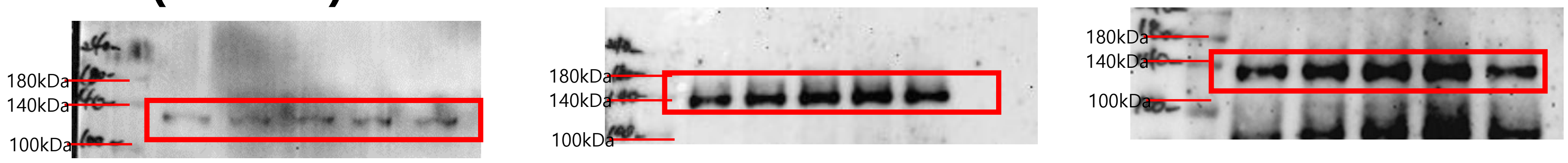

p-pyk2(116kDa)

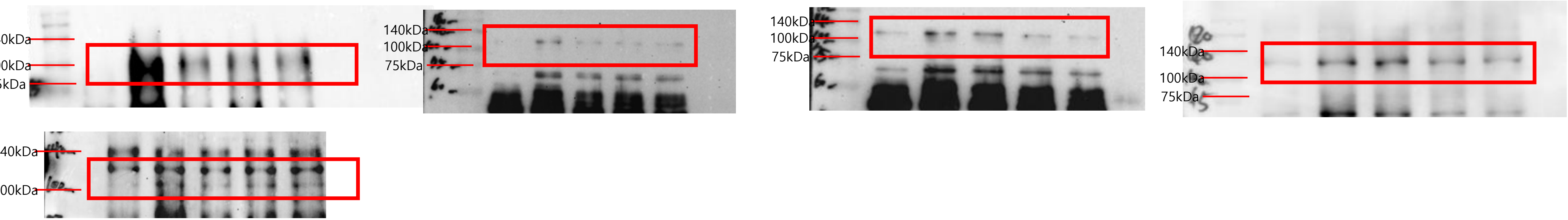

t-pyk2(120kDa)

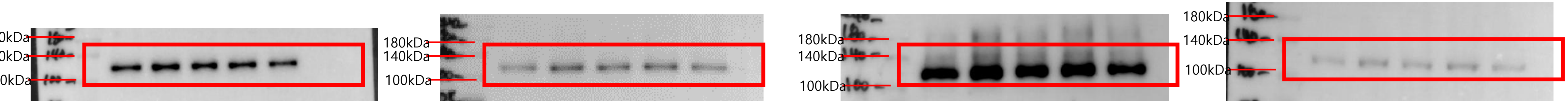

β-actin(43kDa)

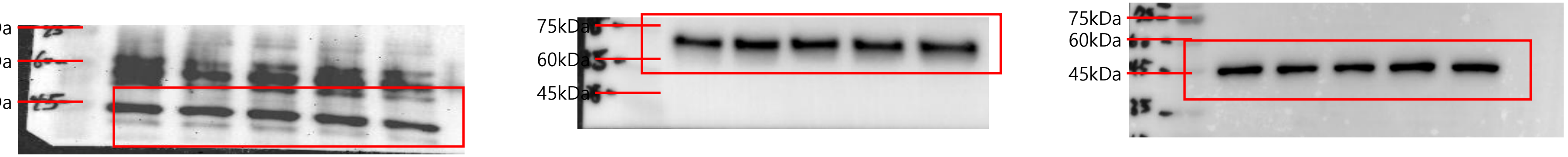

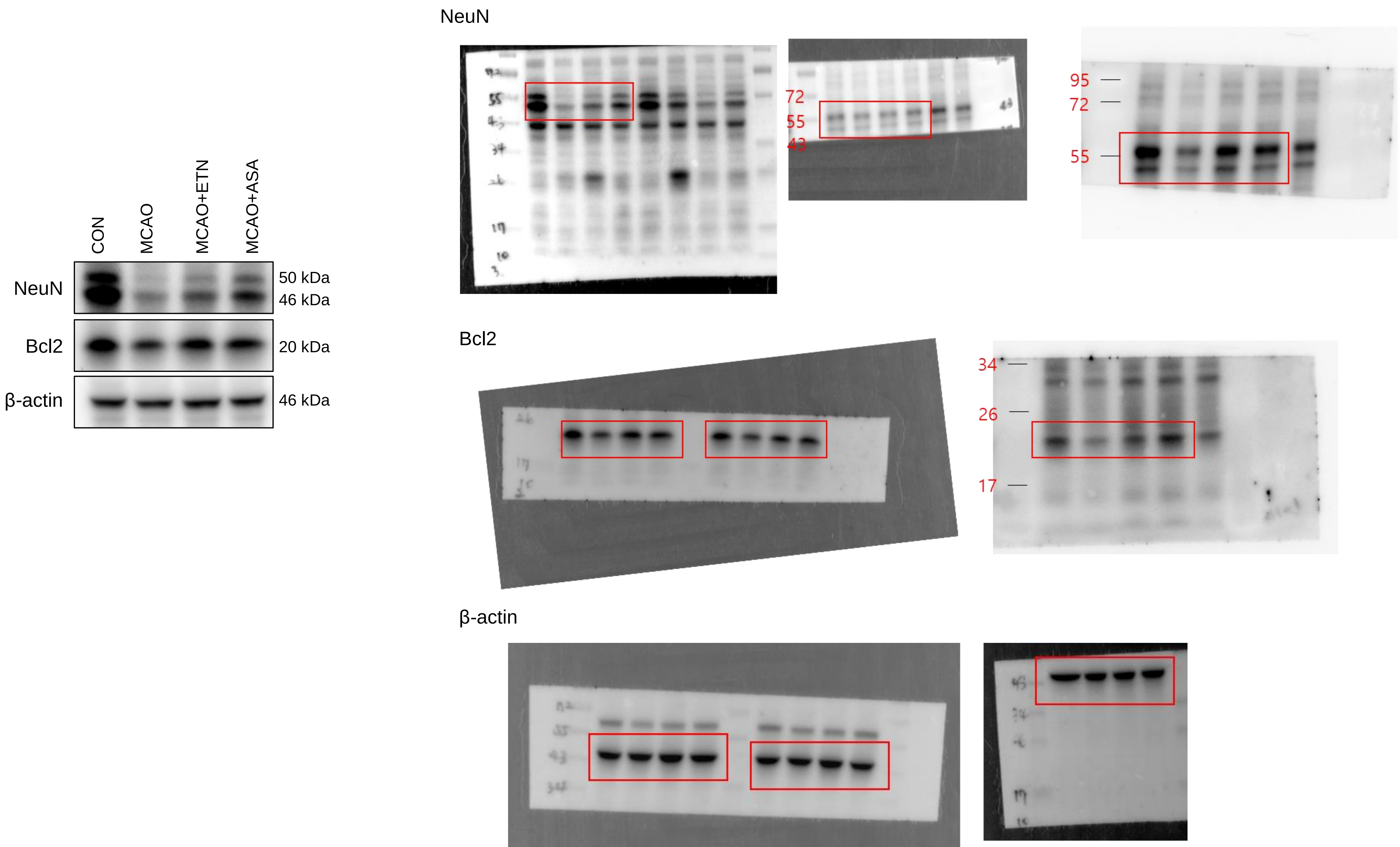
CON
MCAO
MCAO+ETN
MCAO+ASA
NeuN
50 kDa
46 kDa
Bcl2
20 kDa
β-actin
46 kDa
NeuN
72
55
43
95
72
55
Bcl2
34
26
17
β-actin
