# Supplementary material for: The inhibitory effect of gadolinium-conjugated diflunisal on platelet function through regulating outside-in signaling of αIIbβ3 integrin
Source: Front Pharmacol. 2026 Oct 19;17:1936307. doi: 10.3389/fphar.2026.1936307 (PMC13725699; doi:10.3389/fphar.2026.1936307)
Supplement: Supplementary file 2 [file Supplementaryfile1.docx]

Figure S1

ADP 10 μM

Collagen 1 μg/ml

U46619 1μM

A)

B)

C)

**Figure S1. Role of ETN102 in platelet aggregation induced by numerous agonists.** Washed platelets were preincubated with various concentrations of ETN102 (100, 300, or 500 µM) for 5 minutes at 37°C and then stimulated with 1 µg/ml collagen (A), 10 µM ADP (B), and 1 µM U46619 (C). The representative aggregation traces were obtained from five independent experiments. Data represent the mean ± SD (n = 5).

Figure S2

Thrombin (0.05 U/ml)

**Figure S2. Effects of ETN102 on TxB_2_ generation.** Washed platelets were preincubated with ETN102 at concentrations of 100, 300, or 500 µM for 5 minutes at 37°C. Following stimulation, TxB_2_ production was quantified using a TxB_2_ ELISA assay kit. All data are presented as mean ± SD (n = 5).

Figure S3

Thrombin (0.05 U/ml)

Thrombin (0.05 U/ml)

B)

A)

**Figure S3. ETN102 does not modulate Ca^2+^ mobilization during platelet activation.** Mouse platelets were suspended in HEPES-Tyrode buffer without 1 mM CaCl_2_ and preincubated with ETN102 (100, 300, or 500 µM) for 5 minutes at 37°C. The platelets were then incubated with a calcium-sensitive dye for 30 minutes at 37°C in the dark. Following treatment with a Ca^2+^ dye, the platelets were stimulated with 0.05 U/ml thrombin, followed by the addition of 2 mM CaCl_2_ to induce Ca^2+^ influx. Intracellular Ca^2+^ release (A) and Ca^2+^ influx (B) were recorded and quantified by calculating the area under the curve (AUC, arbitrary units). Quantitative data are presented as mean ± SD (n = 5).

Figure S4

B)

A)

Thrombin

-	+	+	+	+

ETN102

100 300 500

(μM)

Vehicle

+	+

p-Syk

p-PI3K

p-AKT

total-PI3K

total-AKT

total-Syk

**Figure S4. ETN102 does not affect thrombin-induced phosphorylation of Syk, PI3K, and AKT in platelets.** Mouse platelets were pre-treated with ETN102 (100, 300, or 500 µM) and stimulated with thrombin (0.05 U/ml). (A) Representative immunoblots showing the phosphorylation levels of Syk, PI3K, and AKT. (B) Quantitative analysis of phosphorylated Syk, PI3K, and AKT levels. Data are presented as mean ± SD (n = 3).

Figure S5

B)

A)

**Figure S5. Effects of ETN102 on platelet LDH release and HT22 cell viability.** (A) LDH release from washed platelets after incubation with vehicle or ETN102 (100, 300, or 500 µM) for 15 minutes at 37°C. LDH release is expressed relative to the maximum-release control. (B) Viability of HT22 cells after treatment with vehicle or ETN102 (100, 300, 500, or 1,000 µM) for 24 hours, measured using the CCK-8 assay and expressed relative to the vehicle control. Data are presented as mean ± SD (platelet LDH, n = 5; HT22 viability, n = 3). Statistical comparisons were performed using one-way ANOVA followed by Dunnett’s multiple-comparisons test. **:P<0.01, and ***:P<0.001 compared with vehicle control.
